# KI and WU Polyomaviruses: Seroprevalence Study and DNA Prevalence in SARS-CoV-2 RNA Positive and Negative Respiratory Samples

**DOI:** 10.3390/microorganisms10040752

**Published:** 2022-03-30

**Authors:** Melinda Katona, Krisztina Jeles, Renátó Kovács, Eszter Csoma

**Affiliations:** 1 Doctoral School of Pharmaceutical Sciences, University of Debrecen, Nagyerdei Krt. 98, 4032 Debrecen, Hungary; katona.melinda@med.unideb.hu (M.K.); jeleskrisztina@med.unideb.hu (K.J.); 2 Department of Medical Microbiology, Faculty of Medicine, University of Debrecen, Nagyerdei Krt. 98, 4032 Debrecen, Hungary; kovacs.renato@med.unideb.hu; 3 Faculty of Pharmacy, University of Debrecen, Nagyerdei Krt. 98, 4032 Debrecen, Hungary

**Keywords:** polyomaviruses, WUPyV, KIPyV, SARS-CoV-2, DNA prevalence, seroprevalence

## Abstract

The aim of this work was to study the possible co-infection of KI and WU polyomavirus (KIPyV and WUPyV, respectively) and severe acute respiratory syndrome coronavirus 2 (SARS-CoV-2) in respiratory samples and to detect the seroprevalence of KIPyV and WUPyV. A total of 1030 nasopharyngeal samples were analyzed from SARS-CoV-2 RNA positive (*n* = 680) and negative (*n* = 350) adults and children (age: 1 day to 94.2 years) collected from August 2020 to October 2021. KIPyV DNA was detected in two SARS-CoV-2-positive samples (2/680, 0.29%) and in three SARS-CoV-2-negative samples (3/350, 0.86%). WUPyV DNA was observed in one-one samples from both groups (1/680, 0.15% vs. 1/350, 0.29%). We did not find an association between SARS-CoV-2 and KIPyV or WUPyV infection, and we found low DNA prevalence of polyomaviruses studied after a long-term lockdown in Hungary. To exclude a geographically different distribution of these polyomaviruses, we studied the seroprevalence of KIPyV and WUPyV by enzyme-linked immunosorbent assay among children and adults (*n* = 692 for KIPyV and *n* = 705 for WUPyV). Our data confirmed that primary infections by KIPyV and WUPyV occur mainly during childhood; the overall seropositivity of adults was 93.7% and 89.2% for KIPyV and WUPyV, respectively. Based on our data, we suggest that the spread of KIPyV and WUPyV might have been restricted in Hungary by the lockdown.

## 1. Introduction

In 2007, two new viruses were described within the *Polyomaviridae* family. KI (Karolinska Institute) polyomavirus (KIPyV) and WU (Washington University) polyomavirus (WUPyV) were detected in respiratory samples from children with acute respiratory symptoms [1,2]. Although these viruses might be transmitted via the respiratory route and might be respiratory pathogens, their pathogenic role has not yet been clarified. Based on seroprevalence studies, these viruses are thought to be ubiquitous in the human population since seropositivity of adults was ≥55% and ≥69% for KIPyV and for WUPyV, respectively [3,4,5,6,7,8]. KIPyV and WUPyV DNA were observed by PCR in different sample types, such as stool, urine, blood, cerebrospinal fluid, and secondary lymphoid tissues. However, respiratory samples were studied the most. Both viruses were found mainly in respiratory samples from children and immunocompromised patients. The DNA prevalence studies with respiratory samples from patients with or without respiratory symptoms resulted in a detection rate up to 12.14% for KIPyV and 0–16.4% for WUPyV [9,10,11,12,13]. Besides viral DNA, WUPyV and KIPyV antigens were also observed in cells of respiratory samples [14,15,16]. In fact, Wang et al. [12] isolated WUPyV and then replicated it successfully in vitro. In some cases, KIPyV and WUPyV were revealed as the only potential causative agent of respiratory diseases, but in many studies the co-infection rates with other pathogens were high [9,17,18]. Accordingly, WUPyV and KIPyV might also be opportunistic pathogen as co-infectious viruses.

Severe acute respiratory syndrome coronavirus 2 (SARS-CoV-2) can cause symptomless or mild to severe infection, and even fatal coronavirus disease 19 (COVID-19). Co-infection and superinfection of patients with other respiratory pathogens, such as bacteria, fungi, and viruses have been published, and these co-detected pathogens might affect the outcome of SARS-CoV-2 infection [19]. Little is known about the prevalence of WU and KI polyomaviruses related to SARS-CoV-2 infection. So far, only Prezioso and colleagues [20] have published data: 24.1% KIPyV and 4.5% WUPyV co-infection in respiratory samples from patients infected with SARS-CoV-2.

Our aim was to study the prevalence of KIPyV and WUPyV DNA in nasopharyngeal samples from patients who had tested positive or negative for SARS-CoV-2 RNA. Since there are no KIPyV and WUPyV seroprevalence data from Hungary, we also performed antibody detection to compare the geographical distribution of these viruses.

## 2. Materials and Methods

The study was approved by Regional and Institutional Research Ethics Committee, Clinical Centre, University of Debrecen, Hungary (DE RKEB/IKEB: 5134-2018 and DE RKEB/IKEB: 5770-2021). Samples were analyzed anonymously. Thus, consent from patients was neither required nor obtained.

### 2.1. Serum Samples for Seroepidemiology

Serum samples from children and adults sent for routine diagnostics (Epstein–Barr virus, cytomegalovirus, hepatitis B virus, hepatitis C virus and SARS-CoV-2 serology) to Medical Microbiology, University of Debrecen, Hungary, between 2016 and 2021 were analysed. Samples were stored at −70 °C until usage. The WUPyV enzyme-linked immunosorbent assay (ELISA) was performed by using 705 serum samples (373 from children and 332 from adults), while the KIPyV antibody was screened in 692 samples (325 children and 367 adult sera).

### 2.2. Expression and Purification of Recombinant Proteins

The gene encoding the major capsid protein (VP1) of WUPyV (WU Polyomavirus strain B0, GenBank accession number: EF444549.1) was codon optimized and commercially synthesized (GeneArt Gene Synthesis, Thermo Fisher Scientific, Waltham, MA, USA) with a 6xHis tag at the N-terminus. The VP1 gene of KIPyV (KI polyomavirus Stockholm 60, GenBank accession number: NC_009238.1) was obtained in the same way (GeneArt Gene Synthesis), with a 6xHis tag at the N-terminus but without codon optimization. Both genes were inserted into plasmid pTriEx™-4 Neo (Novagen, Pretoria, South Africa, Merck, Kenilworth, NJ, USA). *Escherichia coli* Origami™ B(DE3)pLacI (Novagen, Pretoria, South Africa) were transformed with the recombinant vector containing the WUPyV VP1 gene, while *E. coli* Rosetta-gami™ B(DE3)pLacI were transformed with the vector containing the KIPyV VP1 gene. Recombinant viral proteins were expressed after induction and then affinity purified under denaturing condition using Protino^®®^ Ni-TED Packed Columns (Macherey-Nagel, Düren, Germany) according to the manufacturer’s instruction. Urea was removed by dialysis using a 10 kDa Slide-A-Lyzer Dialysis cassette (Themo Fisher Scientific, Waltham, MA, USA). Following concentration with a 30 kDa Amicon Ultra centrifugal filter (Merck, Kenilworth, NJ, USA), protein purity was analyzed Coomassie brilliant blue staining after SDS-PAGE (sodium dodecyl sulphate–polyacrylamide gel electrophoresis) and by western blotting. The primary antibody was against the 6xHis tag (mouse monoclonal anti-HIS antibody, Thermo Fisher Scientific, Waltham, MA, USA). The secondary antibody was goat anti-mouse, superclonal conjugated to horseradish peroxidase (HRP) (Thermo Fisher Scientific, Waltham, MA, USA). The protein concentration was determined by Pierce BCA Protein Assay kit (Thermo Fisher Scientific, Waltham, MA, USA).

### 2.3. ELISA and Cut-Off Determination

An indirect ELISA was developed, including determination of the optimal coating concentration, blocking buffer and concentration, dilutions of primary and secondary antibodies, incubation time and temperature and washing buffer and cycles. Briefly, the wells of MaxiSorp 96-well plates (Nunc) were coated with purified VP1 protein (50 ng for KIPyV and 100 ng for WUPyV in 100 µL/well) overnight at 4 °C in ELISA/ELISPOT Coating Buffer (phosphate-buffered saline, PBS), pH 7.4, Thermo Fisher Scientific, Waltham, MA, USA). The wells were washed twice with PBS, then blocked with 2% casein (Sigma, St. Louis, MI, USA) in PBS for 1 h at 37 °C. After washing each well twice with PBS, 100 µL of each serum sample diluted was added to each well. The dilution (1:100) was performed in PBS containing 0.05% Tween-20 (PBS-T). Following the incubation for 1 h at 37 °C, and then three times wash steps with PBS-T, HRP-conjugated secondary antibody (goat anti-human IgG Fc Highly Cross-Adsorbed Secondary Antibody, Thermo Fisher Scientific, Waltham, MA, USA) diluted 1:10,000 in PBS-T was added; the plate was incubated at 37 °C for 1 h. After washing each well three times with PBS-T, 3,3′,5,5′-tetramethylbenzidine (TMB) substrate was added; the plate was incubated for 15 min at room temperature. 1 M H_2_SO_4_ was used to stop the enzyme reaction, and then the absorbance at 450 nm was measured by using a Multiscan Sky Microplate Spectrophotometer (Thermo Fisher Scientific, Waltham, MA, USA). All samples were measured in duplicate and the average value optical density (OD) was determined (the blank value was subtracted from the OD of each sample).

The cut-off value without positive and negative controls was determined by first plotting the ranked OD values and drawing a tendency curve. Then, the inflection point was calculated based on polynomial regression [21,22].

### 2.4. Samples for the DNA Prevalence Study

Nasopharyngeal swab samples sent for SARS-CoV-2 PCR from August 2020 to April 2021 and in October 2021 to Medical Microbiology, University of Debrecen, Hungary, were analyzed. Nucleic acid was extracted by using the MagNA Pure 96 DNA and Viral NA Small Volume Kit (Roche, Basel, Switzerland) or the Chemagic Viral DNA/RNA 300 Kit H96 (PerkinElmer, Turku, Finland) according to the manufacturers’ instructions. SARS-CoV-2 RNA was detected by using the ViroReal^®^ Kit SARS-CoV-2 & SARS (Ingenetix GmbH, Wien, Austria) or the SARS-CoV-2 RT-qPCR Reagent Kit (PerkinElmer, Turku, Finland) according to the recommended protocol. Table 1 summarizes the patient data. Out of the 1030 samples 350 samples were collected from children (age range: 0–17.8 years; median: 8.2 years). Out of the 350 children 148 were SARS-CoV-2 negative and 202 were SARS-CoV-2 positive, respectively.

### 2.5. Viral DNA Detection

A multiplex real-time PCR was applied to detect KIPyV and WUPyV DNA as detailed previously [23]. The template nucleic acid was 10 µL in 50 µL reaction volume [24]. 

### 2.6. Statistical Analysis

Statistical analyses were performed by using the nonparametric Fisher’s exact test and the Mann–Whitney test. A *p* value < 0.05 was considered significant.

## 3. Results and Discussion

### 3.1. Seroprevalence of KIPyV and WUPyV

**Figure 1:**
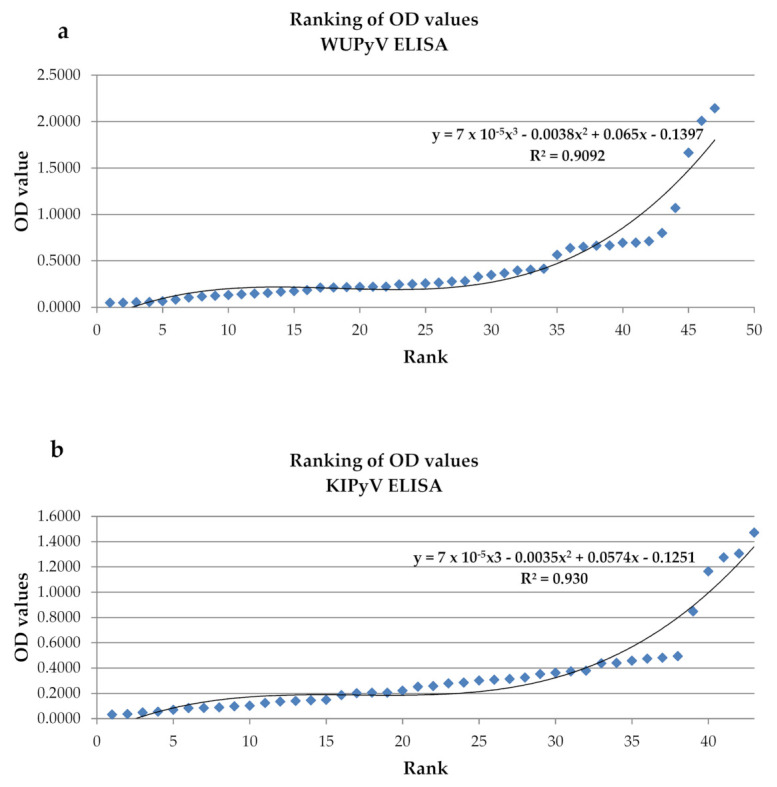
Ranking the optical density (OD) values of patients <3 years old measured in the (**a**) KIPyV and (**b**) WUPyV enzyme-linked immunosorbent assay (ELISA). Diamonds represent each OD value. The polynomial trend line is the line of best fit; the correlation coefficient (R^2^) and the function used to calculate the inflection point are indicated. KIPyV, KI polyomavirus; WUPyV, WU polyomavirus.

To investigate past infection caused by WUPyV and KIPyV in our study population, IgG against the VP1 proteins of the viruses was detected in serum samples using indirect ELISA. The seroprevalence rate was calculated as the proportion of the sera that displayed an OD value above the determined cut-off. This cut-off value was determined by using the OD value of children <3 years, since based on the available data, a high proportion of children within this group acquire the infection [3,21,25]. Figure 1 shows the plots of the OD values and the tendency curves used to determine the inflection points. To determine the positivity, the cut-off value was calculated as the OD value based on the inflection point +10% grey zone. Based on the above-mentioned criteria, samples with >0.21 OD and >0.23 OD were considered seropositive for KIPyV and WUPyV, respectively.

The overall seropositivity in this study was 82.1% (568/692) for KIPyV and 79.1% (558/705) for WUPyV. At the same time, the adult seropositivity was 93.7% for KIPyV and 89.2% for WUPyV. The sex distribution of the patients seropositive for KIPyV and WUPyV was not different from the total group of the patients. Moreover, there was no difference between the children and adult groups studied (Fisher’s exact test, *p* > 0.05). 

The age of the seropositive patients was significantly higher than the age of the total population studied for both KIPyV (Mann–Whitney test, *p* = 0.005) and WUPyV (Mann-Whitney test, *p* = 0.02). There was a statistically significant age difference between seropositive and seronegative patients within the cohort, and also within the group of children for both viruses, but not within the group of adults (Mann–Whitney test, *p* values indicated in Table 2).

**Figure 2:**
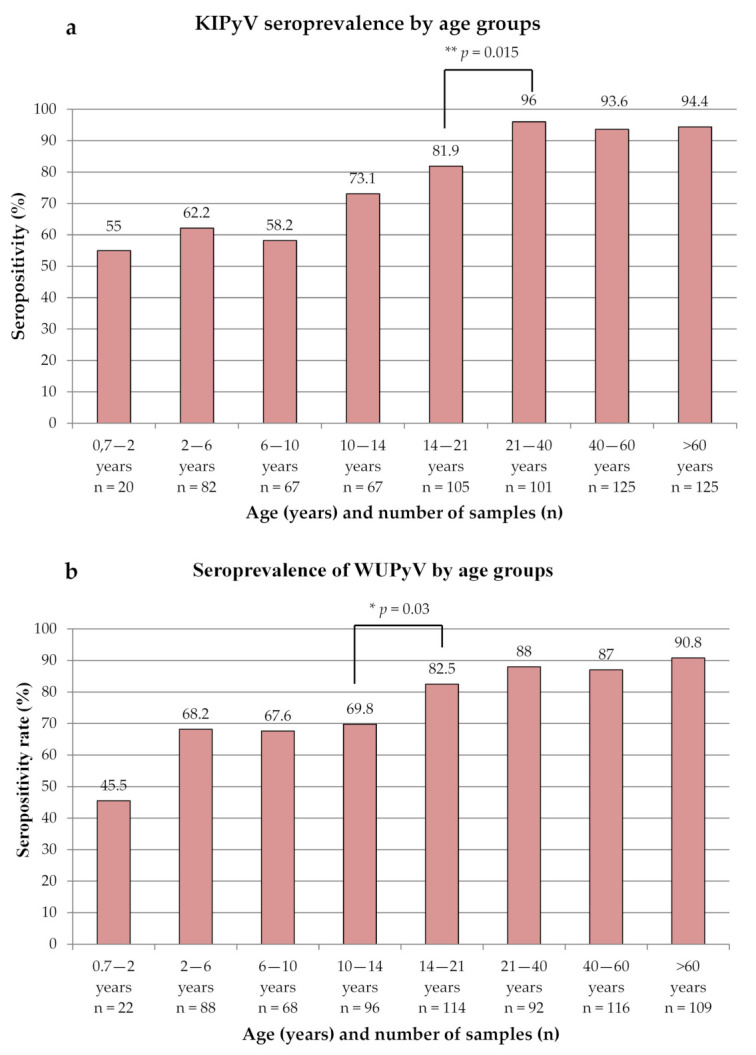
Age distribution of seropositivity for KI polyomavirus (KIPyV) (**a**) and WU polyomavirus (WUPyV) (**b**) seropositivity rates by age groups.

In accordance with data published by others, our seroprevalence results suggest that primary infection by both KIPyV and WUPyV occurs during childhood. We detected 55% and 45.5% seropositivity for patients under two years of age for KIPyV and WUPyV, respectively [3,5,21,26]. Similarly to Neske et al. [5], there was a higher prevalence of KIPyV IgG in children, although we detected the highest seropositivity rate (96%) in the age group of 21–40 years. There was a significant increase in the seropositivity rate from 81.9% to 96% between the age groups of 14–21 years and 21–40 years (Fisher’s exact test, *p* = 0.0015), but then no other significant changes. In the case of WUPyV, seropositivity reached ~68% in the age group of two to six years, followed by no additional changes until 14 years. There was a significant increase in WUPyV IgG prevalence between the age group of 10–14 years and 14–21 years (Fisher’s exact test, *p* = 0.034). The seroprevalence reached the maximum of 90.8% in the age group of >60 years. The data are presented in Figure 2.

**Figure 3:**
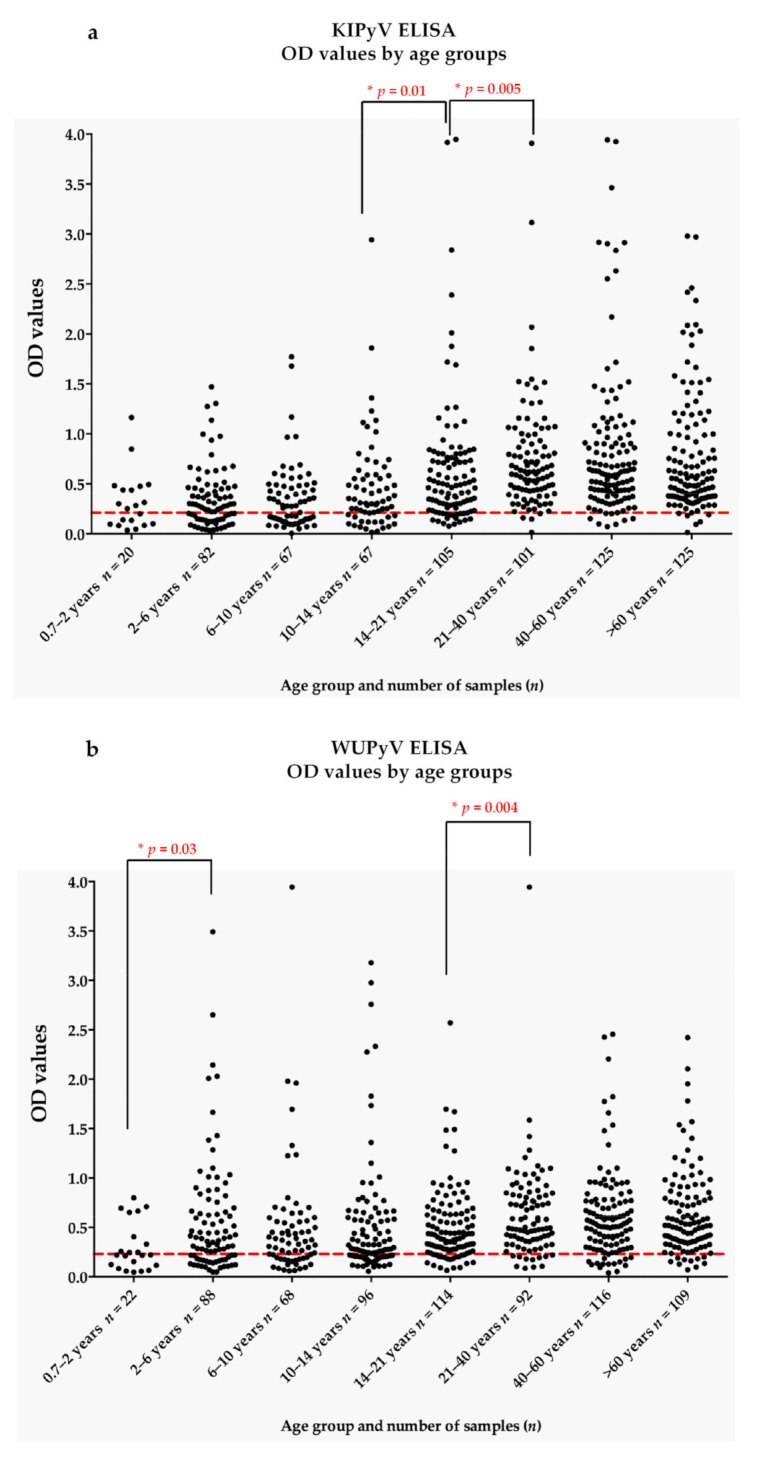
Age distribution of optical density (OD) values measured in the KI polyomavirus (KIPyV) (**a**) and WU polyomavirus (WUPyV). (**b**) enzyme-linked immunosorbent assay (ELISA). The red dashed line represents the cut-off value above which the seropositivity determined.

For the KIPyV ELISA, there was a significant difference in OD values between the age groups of 10–14 years and 14–21 years (Mann–Whitney test, *p* = 0.01), and also between the age groups of 14–21 years and 21–40 years (Mann–Whitney test, *p* = 0.005) (Figure 3a). For the WUPyV IgG ELISA, there was a significant difference in OD values between the age groups of 0.7–2 years and 2–6 years (Mann–Whitney test, *p* = 0.03), and also between the age groups of 14–21 years and 21–40 years (Mann–Whitney test, *p* = 0.004) (Figure 3b).

### 3.2. DNA Prevalence of KIPyV and WUPyV

In total, 1030 nasopharyngeal swab samples were tested for the presence of KI and WU polyomavirus DNA. The age and sex of the patients did not differ between the SARS-CoV-2-positive (*n* = 680) and SARS-CoV-2-negative (*n* = 350) specimens. KIPyV DNA was detected in 5/1030 (0.49%) samples of which 2/680 (0.29%) were SARS-CoV-2-positive samples and 3/350 (0.86%) were SARS-CoV-2-negative samples. The WUPyV DNA prevalence was 2/1030 (0.19%): positivity was 1/680 (0.15%) for SARS-CoV-2-positive samples and 1/350 (0.29%) for SARS-CoV-2-negative samples. Table 3 summarizes the data. Our prevalence data revealed markedly lower KIPyV and WUPyV positivity in SARS-CoV-2-positive patients than published by Prezioso et al. [20]. Both KI and WU polyomaviruses have rarely or not been identified in respiratory samples from immunocompetent adults; they have mainly been described in respiratory samples from immunocompetent and immunocompromised children, as well as immunocompromised adults [1,24,27,28,29,30]. Hence, it is important to analyze our samples in more detail.

We previously found 1.4% KIPyV and 4.1% WUPyV DNA positivity in samples from 2.5–12.1-year-old children (*n* = 146) [23]. In the present study, out of the children cohort 138 samples were selected from the age range 2.5–12.1 years (median 6.2 years), without an age difference compared with the previous study (Mann–Whitney test, *p* = 0.49). In the present study, KIPyV was not detected in any samples from this cohort of children, and only one specimen was WUPyV DNA positive (1/350, 0.29%), collected from a SARS-CoV-2-positive child (1/202, 0.49%). When analyzing our previous and present data, there was no difference for WUPyV positivity among 2.5–12.1-year-old children (6/146 vs. 1/138; Fisher’s exact test, *p* = 0.1214), and there was no difference for KIPyV positivity (2/146 vs. 0/138; Fisher’s exact test, *p* = 0.4986). Prezioso et al. [20] did not study samples from children.

In a previous study, we applied the same real-time PCR method and did not detect KI and WU polyomaviruses in respiratory samples of immunocompetent adults, non-pregnant and pregnant women [31]. On the other hand, we detected KIPyV and WUPyV DNA in 14.3% and 9.1%, respectively, of the respiratory samples from immunocompromised, kidney transplant patients [32]. In the adult group of the patients in this study (*n* = 680, 18–94.2 years, median 56.5 years), 5/680 (0.74%) samples were positive for KIPyV DNA; there was no difference between SARS-CoV-2-positive and SARS-CoV-2-negative patients (2/478 vs. 3/202; Fisher’s exact test, *p* = 0.1584). WUPyV DNA positivity was observed in one specimen of a SARS-CoV-2-positive adult (1/680; 0.15%). Neither KIPyV nor WUPyV DNA positivity was detected in samples from SARS-CoV-2-negative patients (0/38) in the other study [20] investigating SARS-CoV-2 and polyomavirus co-infection. At the same time, we found difference compared with Prezioso et al. [20]: our KIPyV DNA prevalence was significantly lower in samples from SARS-CoV-2-positive adult patients (2/478 vs. 27/122; Fisher’s exact test, *p* = 0.0001), and WUPyV positivity showed significant difference in SARS-CoV-2-positive adult patients (1/478 vs. 5/112; Fisher’s exact test, *p* = 0.0012). 

The reason for the differences can be explained by several factors. We used the same real-time PCR method as Prezioso et al. [20]. Although the nucleic acid isolation kits are different, we have used the same DNeasy Blood and Tissue Kit (Qiagen) used by Prezioso et al. for respiratory samples of children, and we did not find statistically significant difference between data of that study and the present one [20,23]. In our investigation, nasopharyngeal samples were collected, while we had previously analyzed throat swabs [23,31,32,33]. Prezioso et al. [20] examined oropharyngeal samples. At the same time, various samples types—nasopharyngeal, oropharyngeal, nasal and throat swabs—have been used successfully to detect the polyomaviruses [9]. In the present, retrospective study, we have no information on the immunological status of the patients. Likewise, Prezioso et al. [20] did not publish such data. Immunosuppression may result in higher susceptibility to infection or may strengthen reactivation if the viruses establish latency. Since our seroprevalence data (detailed above) are in accordance with data published by others, geographical differences are not obvious.

When and what government restrictions were in place due to the COVID-19 pandemic may have significantly influenced the spread of the viruses. In March 2020 in Hungary, the government declared a state of emergency due to the COVID-19 pandemic, which was followed by a number of restrictive measures and recommendations for social distancing. In Italy, the lockdown also started in March 2020. Prezioso et al. [20] analyzed samples collected from March to May 2020, while our samples were collected from August 2020, after a long-term lockdown. Lockdown, curfew, restricted use of different services, school, and day care closure and social distancing had great impacts on the spread of not only SARS-CoV-2, but also other pathogens [34]. In addition, close contacts and social activities of children decreased markedly in Hungary. Although we had samples from the period with somewhat relaxed restrictions (August–October 2020 and October 2021), the spread of the respiratory pathogens may not have occurred immediately despite re-opening.

## 4. Conclusions

In the present study, there was no association between SARS-CoV-2 and KI and WU polyomavirus infections. Despite the higher number of respiratory samples, our data are significantly different from the data published by an Italian research team [20]. Since we detected similar seropositivity rates of these polyomaviruses in Hungary as published by others, KIPyV and WUPyV infections are also ubiquitous in Hungary. The respiratory samples analyzed in our work were collected after a long-term lockdown due to the COVID-19 pandemic, a factor that might explain the differences in co-infection rates.

## Figures and Tables

**Table 1:** Data of patients studied for WU and KI polyomavirus prevalence.

	Number of Samples	Age in Years, Min–Max (Median)	Female/Male
SARS-CoV-2-positive patients	680	0.1–94.2 (35.7)	353/327
SARS-CoV-2-negative patients	350	0–94.1 (48.9)	174/176
Total	1030	0–94.2 (38.1)	527/503

**Table 2:** Detailed data of patients seropositive and seronegative for WU and KI polyomavirus.

	Patient Group	Age in Years, Min–Max (Median); Mean	Female/Male	Seropositivity
KIPyV ELISA	Total (*n* = 692)	0.7–92 (21.9); 31.3	343/349	82.1%
	P (*n* = 568)	0.7–92 (30.8); 34.9 * *p* = 0.005	284/284	
	N (*n* = 124)	0.7–81 (8.80); 15.1 *** *p* = 0.0001	59/65	
	Children (*n* = 325)	0.7–17.9 (9.60); 9.37	156/169	68.9%
	P (*n* = 224)	0.7–17.9 (11.0); 10.1	111/113	
	N (*n* = 101)	0.7–19 (7.00); 7.74 ** *p* = 0.0002	45/56	
	Adult (*n* = 367)	18–92 (50.2); 50.8	187/180	93.7%
	P (*n* = 344)	18–52 (50.4); 51.0	173/171	
	N (*n* = 23)	19–81 (43.8); 47.3	14/9	
WUPyV ELISA	Total (*n* = 705)	0.7–92 (16.8); 28.7	353/352	79.1%
	P (*n* = 558)	0.7–92 (21.5); 31.5 * *p* = 0.02	298/260	
	N (*n* = 147)	0.7–81 (11.3); 18.0 *** *p* = 0.0001	55/92	
	Children (*n* = 373)	0.7–17.9 (10.4); 9.62	185/188	70.2%
	P (*n* = 262)	0.7–17.9 (11.0); 10.1	143/119	
	N (*n* = 111)	0.7–17.9 (7.90); 8.45 *** *p* = 0.004	42/69	
	Adult (*n* = 332)	18–92 (50.1); 50.1	168/164	89.2%
	P (*n* = 296)	18–92 (50.6); 50.4	155/141	
	N (*n* = 36)	21.7–81 (45.7); 47.5	13/23	

The red color indicates which groups were analyzed and showed a statistically significant difference; the corresponding *p* value is included. Abbreviations: ELISA, enzyme-linked immunosorbent assay; KIPyV, KI polyomavirus; WUPyV, WU polyomavirus; P, seropositive; N, seronegative; n: number of patients.

**Table 3:** Detailed data of patients studied for KIPyV and WUPyV DNA prevalence.

	SARS-CoV-2-Positive Patients			SARS-CoV-2-Negative Patients		
	Number of Samples	Age in Years, Min–Max (Median)	Female/Male	Number of Samples	Age in Years, Min–Max (Median)	Female/Male
KIPyV DNA positive	2	26.6 and 72.8	1/1	3	18.3; 50.1 and 72.8	3/0
KIPyV DNA negative	678	0.1–94.2 (35.7)	352/326	347	0–94.1 (48.8)	171/176
WUPyV DNA positive	1	36.2	1/0	1	3.7	1/0
WUPyV DNA negative	679	0.1–94.2 (35.6)	352/327	349	0–94.1 (49)	173/176

Abbreviations: KIPyV, KI polyomavirus; WUPyV: WU polyomavirus; SARS-CoV-2: severe acute respiratory syndrome coronavirus 2.

## Data Availability

The data are contained within the article.
